# Study of 18 months of follow up dir floortime intervention in preschool children with autism spectrum disorder (ASD)

**DOI:** 10.1192/j.eurpsy.2021.1346

**Published:** 2021-08-13

**Authors:** P. Pacheco, M. Pacheco, D. Molini-Avejonas

**Affiliations:** 1 Rehabilitation Science, USP, São Paulo, Brazil; 2 Morphology, UFES, Vitória, Brazil

**Keywords:** autism, Development, DIR/Floortime

## Abstract

**Introduction:**

Children in Autism Spectrum Disorder (ASD) have a qualitative deficit in social interaction. The DIR/Floortime (Stanley Greenspan and Serena Wieder) is based on the Child’s Functional Development, Individual Differences and Relationships (D for development, I for individuality or individual differences and R for relationship), aiming at building the foundations for social, emotional and intellectual skills of children.

**Objectives:**

To determine the results of 18 months DIR/Floortime™ parent training for an additional benefit in encouraging children with ASD climbing the developmental “ladder”.

**Methods:**

The participants are 15 children with ASD aged between 2 and 6 years-old. The follow-up occurs in two private DIR Floortime Model service centers in Brazil. Protocols: Functional Emotional Assessment Scale – FEAS and Functional Emotional Developmental Questionnaire – FEDQ. The participants were followed-up one on one every four months.

**Results:**

At the first month of assessment the children showed lack of self regulation, social interaction and engagement with their parents in a symbolic, sensory and motor play setting. At the following evaluations, they showed increase of social interaction and engagement in the same play setting. Besides the high affect of the parents during the play time promoted a two way purpose communication and behavioral organization.

**Conclusions:**

Children showed a good development of functional and emotional capacities during the study period, demonstrating the effectiveness of the DIR/Floortime model in the intervention.

